# The Role of Subjective Well‐Being in Therapist Responsiveness and Session Evaluation: A 10‐Wave Longitudinal Study

**DOI:** 10.1002/cpp.70269

**Published:** 2026-04-09

**Authors:** Marcin Rzeszutek, Paweł Łowicki, Szymon Szumiał, Magdalena Grabowska, Marcin Bagiński, Maria Ibisz, Justyna Kaczmarczyk, Bartłomiej Żukowski, Agata Urbanowicz, Olha Khmil, Monika Rutkowska‐Michalik, Katarzyna Domagała, Kinga Łapińska, Samanta Rode, Teresa Lichota, Dorota Bubiak, Agnieszka Gierulska, Anna Mazur, Łukasz Sztajerowski, Aneta Kosińska, Piotr Fijewski

**Affiliations:** ^1^ Faculty of Psychology University of Warsaw Poland; ^2^ Faculty of Economic Sciences University of Warsaw Poland; ^3^ Ośrodek JA Warsaw Poland; ^4^ Gestalt Institute Cracow Poland; ^5^ Institute of Political Science University of Wroclaw Poland; ^6^ Ośrodek Pomocy i Edukacji Psychologicznej Intra Warsaw Poland

**Keywords:** generalised estimating equations, session evaluation, subjective well‐being, therapist responsiveness

## Abstract

Our study examined the dynamics of the therapist responsiveness (TR) as well as session evaluation by the therapist (SET) with regard to weekly fluctuations in their subjective well‐being (SWB: satisfaction with life and positive/negative affect) in a 10‐week prospective framework while controlling for sociodemographic and work‐related variables. In all, 296 therapists participated in this longitudinal study. The participants filled, after the session with a client chosen for the study period, the Patients' Experience of Attunement and Responsiveness Scale—Therapist Version, the Session Evaluation Questionnaire and the Satisfaction With Life Scale (with the Positive and Negative Affect Schedule) each week. Generalised estimating equations showed that better SWB of therapists was related to almost all the studied dimensions of TR and SET in the sessions during the period of observation. We also noticed significant differences in these associations when we analysed sociodemographic and work‐related variables. The findings call for a greater focus on the psychological well‐being of therapists, which may be linked to the quality of their work with the clients. The therapist's individual characteristics should be taken more into account in studies on the mechanisms of change in psychotherapy research.

## Summary

- Our study examined the impact of therapists' subjective well‐being (SWB) on the quality of their work with clients.
- Specifically, we examined the relationship between perceived therapist responsiveness (TR) and perceived session evaluation (SET), as well as weekly fluctuations in therapists' SWB (i.e., life satisfaction and positive/negative affect).
- Our study's findings suggest the need for more programs to help therapists cope with work‐related stress and practice self‐care.

## 1 Introduction

The psychological well‐being of therapists is still a much understudied area in psychotherapy research, which has traditionally focused predominantly on clients in psychotherapy rather than on the mental functioning of therapists (see reviews, e.g., Simionato and Simpson 2018; Van Hoy and Rzeszutek 2022). Nevertheless, as Norcross and Lambert (2018) underlined in their concluding report of the third APA meeting on evidence‐based treatment: ‘multiple and converging sources of evidence indicate that the person of the therapist is inextricably intertwined with the outcome of therapy’ (p. 307). Until now, most studies have focused on stable therapist characteristics from session to session, such as sociodemographic variables (e.g., age and gender) or work‐related variables (e.g., workload, work experience and professional supervision; Van Hoy and Rzeszutek 2022). The sole focus on such characteristics means that very little is known about within‐therapist factors—that is, the intrapsychic features that can promote well‐being within the profession (Van Hoy et al. 2023a, 2023b). This issue is highly relevant, as there is evidence that therapists provide better treatment when they care for themselves, namely, their emotional functioning and life satisfaction (Brugnera et al. 2020; Chui et al. 2016; Laverdière et al. 2018, 2019). Also, clients themselves usually choose to work with therapists whom they perceive as satisfied with their lives (Lambert and Barley 2001). Some authors have noted that poor life satisfaction may be negatively linked to a therapist's ability to establish a therapeutic alliance (Rupert and Dorociak 2019; Skovholt and Trotter‐Mathison 2016). Specifically, therapists who are experiencing distress due to a lack of self‐care and poor well‐being may be perceived by clients as disrespectful, judgmental and uninterested in forming a genuine connection (Vybíral et al. 2024).

Additionally, Chui et al. (2016) found that the therapist's emotional well‐being may independently contribute to the process and outcome of therapy sessions. In our study, we refer to the classic theory of subjective well‐being (SWB) operationalised as people's satisfaction with their lives and affective balance (i.e., positive and negative emotional reactions to peoples' life; Diener et al. 1985, 2016).

There is still limited knowledge on *how* specific therapist attitudes, behaviours and intrapsychic characteristics impact the therapeutic alliance over time and thus the success or failure of psychotherapy (Abargil et al. 2025; Fiorentino et al. 2024). While client factors have been widely recognised in that process (e.g., Dahl et al. 2014; Nissen‐Lie et al. 2022; Mulder et al. 2017; Watson and Wiseman 2021), therapists' perspectives are much less explored (Lingiardi et al. 2018; Wampold and Owen 2021). To measure such phenomena, several authors have recommended single session analysis, which may provide a bridge between micro‐ and macro‐level mechanisms responsible for long‐term therapy (in)effectiveness (Gelo and Manzo 2015). In our study, we investigated the perceived therapist responsiveness (TR) and the perceived session evaluation by the therapist (SET) with regard to the therapists' SWB with a 10‐week prospective framework.

There is as yet no consensus on how to define TR, as the operationalisations of this construct have substantially evolved over time (for reviews, see Calaboiça et al. 2024; Esposito et al. 2024). One of the first theoretical analysis on TR was performed by Kohut (1971), who described it as the therapist's ability to create a balance during the session by providing enough responses according to the moment in the current therapeutic process. Later, Elkin et al. (2014) suggested a more specific definition in which TR entails the therapist's attentive, affirming and respectful attitude toward the client and demonstrates true interest by responding to the client's communication in the session. More recently, Snyder and Silberschatz (2017) recommended defining TR as the therapist's capacity to be *attuned* to the client's current state during the session. The authors proposed the term *attuned responsiveness* and argued that one should stop perceiving TR from the separate ends of continuum (see optimal vs. nonoptimal TR process; Hatcher 2015), as TR is a fluid construct depending not only on the client's characteristics but also on various intrapsychic characteristics of the particular therapist. Overall, while TR is assumed to facilitate the therapeutic alliance, client engagement and treatment outcomes, it also poses a substantial challenge for psychotherapy research (Stiles and Horvath 2017). First, due to the theoretical overlap of TR with similar constructs (e.g., therapist flexibility or empathy), some authors have employed measurement tools not devoted to assessing this particular concept, which poses risk measurement biases (Esposito et al. 2024). Second, there is also a high dominance of cross‐sectional studies on TR, which precludes causal analysis of this highly changeable phenomenon and its predictors (Calaboiça et al. 2024). Finally, the vast majority of studies have predominantly adopted the client's perspective, so again, the sole therapist outlook in assessing this construct is much neglected (Calaboiça et al. 2024; Esposito et al. 2024).

Apart from TR, another important indicator of the quality of a psychotherapy session is how it is evaluated by the therapists (Fiorentino et al. 2024). More specifically, during negative sessions, which therapists perceived as shallow or worthless, they usually address the client's topics superficially, with little focus on their emotions. In contrast, good sessions, perceived as deep and valuable, create an environment for better exploration of the client's problems. It has been observed that TR is systematically related to the SET of the therapist during the session, and they both predict forming a positive therapeutic alliance over time (Hatcher 2021; Mallinckrodt and Jeong 2015). In particular, the more positive the therapist's perception of the session (see SET), the better their capacity to attune to the client's current state during the session (see TR; Fiorentino et al. 2024). Moreover, numerous authors have observed that the more positive session evaluations are linked with more positive treatment outcomes, which suggests the need for further examination of therapist factors that might facilitate or hinder this in‐session process (Fiorentino et al. 2024).

Finally, there is also some evidence regarding the impact of sociodemographic and work‐related factors on the psychological well‐being of therapists and their capacity to establish a constructive therapeutic alliance. More specifically, a recent systematic review by Van Hoy and Rzeszutek (2022) showed that therapists' well‐being may largely depend on the therapists' age. That is, younger therapists usually tend to experience higher levels of work‐related stress and lower levels of emotional well‐being in comparison to older and more experienced colleagues in that profession. It is often explained by the tendency of some young therapists to have too unrealistic expectations about their roles in this job. In addition, young and less‐experienced therapists, especially those still in training, may also have a weaker ability to mentalise their own emotions, which may be negatively linked to developing the therapeutic alliance (Goldberg et al. 2016; Reading et al. 2019). Moreover, out of work‐related factors, personal therapy occurred to be linked with the better well‐being of therapists (Van Hoy and Rzeszutek 2022). Not only does personal therapy facilitate resolving therapists' painful feelings or inner conflicts, but it also stimulates greater self‐awareness and self‐reflection (Moe and Thimm 2021; Orlinsky et al. 2005, 2011). Personal therapy may also act as a buffer against burnout in this profession (Deighton et al. 2007; Garcia et al. 2018; Wiseman and Egozi 2006). This last finding aligns with existing evidence showing that the vast majority of therapists (>80%) report that personal therapy is helpful and contributes positively to their professional development (Orlinsky et al. 2005). Finally, some authors also found differences between therapeutic modalities, with respect to the TR, but this topic is still highly understudied and needs more empirical investigation (Davì et al. 2024).

### 1.1 Current Study

Taking the above‐mentioned research gaps into consideration, the main aim of this study was to examine the dynamics of subjectively perceived TR and the perceived SET with regard to weekly fluctuations in the therapists' SWB (satisfaction with life and positive/negative affect; PA/NA), while controlling for sociodemographic and work‐related variables in a sample of therapists. More specifically, our goal was to examine whether perceived TR and SET were more optimal in weeks when therapists' SWB was higher. The opposite trend could be assumed during weeks when participants reported poor SWB. To the best of our knowledge, no studies have been conducted on the mutual link between these sets of variables using our data analytical strategy and this prospective methodological design. Thus, our study is mainly explorative. However, based on previous studies on psychological well‐being among therapists (e.g., Laverdière et al. 2018,; Van Hoy et al. 2023a, 2023b), we formulated two general hypotheses:

1. On weeks with better SWB (i.e., high life satisfaction, high PA and low NA), therapists are characterised by higher perceived TR during the session, as operationalised by the degree of their helpfulness and the scope of acceptance of the client. The opposite trend can be observed during weeks with poor SWB among participants.
2. On weeks with better SWB, therapists are characterised by higher perceived SET during the session, as operationalised by the degree of perceived depth, smoothness, positivity and arousal during the session. The opposite trend can be seen during weeks with poor SWB among participants.

## 2 Method

### 2.1 Participants

The recruitment procedure consisted of sending the online invitations to the study through five professional psychotherapeutic associations and six psychotherapy schools, representing the main psychotherapy approaches in Poland (i.e., psychoanalytic‐psychodynamic, humanistic‐existential, cognitive‐behavioural, systemic and integrative). We also sent these invitations to the individual psychotherapy offices, using personal professional networks, in order to gather the potentially highest baseline sample of therapists. In all, 296 therapists aged 25–77 (M = 43.23; SD = 8.40) years participated in the study: 250 women aged 25–69 (M = 42.97; SD = 8.18) and 46 men aged 28–77 (M = 44.61; SD = 9.45). The work experience of the therapists fell within the range of 1 to 52 years (M = 8.01; SD = 7.67). Table 1 presents the characteristics of the sample regarding sociodemographic and work‐related variables.

**TABLE 1:** Sociodemographic and work‐related variables.

Variable	*n*	%	Variable	*n*	%
Gender			Therapeutic approach		
Female	250	84.5	Humanist‐existential	183	61.8
Male	46	15.5	Integrative	39	13.2
Marital status			Psychodynamic	34	11.5
Single	38	12.8	Cognitive‐behavioural	15	5.1
Stable relationship	217	73.3	Psychotraumatology	10	3.4
Divorced	41	13.9	Systemic	8	2.7
Education			Other	7	2.4
Psychology	190	64.2	Therapeutic certificate		
Social sciences	54	18.2	Yes	124	41.9
Medical or STEM	12	4.1	In training	161	54.4
Humanities	31	10.5	Not applicable	11	3.7
No information	9	3.0			
Patients			Supervision		
Adults	291	98.3	Once a week	95	32.1
Children and teens	86	29.1	Once a month	188	63.5
Couples	69	23.3	Once a quarter or less often	13	4.4
Families	25	8.4	Own therapy		
Work status			Yes	217	73.3
Private practice	284	95.9	No	5	1.7
Public sector	62	20.9	In therapy	74	25.0
Volunteering	8	2.7			
Other	18	6.1			

The majority of the participants were in stable relationships, had a background in psychology and worked with adult patients in private practice. The humanistic‐existential approach was the most common therapeutic approach among the participants. The majority of participants were undergoing psychotherapeutic training. Their work was supervised once a week. The majority of study participants had completed their own psychotherapy.

### 2.2 Procedure

The study used a 10‐week longitudinal design in order to track the dynamics of the participants' perceptions of TR and SET with regard to their weekly fluctuations in SWB. The inclusion criteria for our study encompassed a minimum of 1 year of clinical experience and the ability to regularly meet with the same selected client over a period of 10 weeks. Participants were recruited through centres for psychotherapeutic training and practice all over Poland.

Therapists were instructed to select one client at the beginning of the study, preferably a client with whom they felt they had established a therapeutic relationship and who was expected to continue the therapeutic process for the duration of the study. The weekly set of measures included TR and SET, along with SWB tools (see Measures). In addition, the participants completed demographic and work‐related questions in Weeks 1 and 10. All questionnaires were administered online via customised survey links. Each week, participants had time from 9 am on Monday until the end of Friday to fill out the questionnaire, although they were encouraged to do so shortly after the session whenever possible. They were asked to respond to the questions in reference to the same client each week. In cases when the session was missed or cancelled for any reason, therapists were asked to fill in the measures in reference to the most recent session with this client. Reminders were sent twice a week to encourage timely completion of the questionnaires. The research team tracked submissions weekly and followed up with nonrespondents to minimise missing data. All participants provided written informed consent to take part in this study and participated in it without remuneration. The study received a positive opinion from the local ethics committee.

### 2.3 Measures

#### 2.3.1 Patients' Experience of Attunement and Responsiveness Scale—Therapist Version

The Polish version of the Patients' Experience of Attunement and Responsiveness Scale—Therapist Version (PEAR‐T; Snyder and Silberschatz 2017) was used to assess the perceived therapists' responsiveness among the study participants. It consists of 22 self‐reported items rated on a 5‐point Likert scale ranging from 0 (not at all) to 4 (very much). Two dimensions are calculated: *therapist helpfulness* (describing therapists' perceptions of the extent to which a patient considered their attitude and interventions useful; e.g., ‘I was able to provide valuable insight to my client that resulted in him/her achieving greater self‐understanding today’) and *safe–accepted* (indicating therapists' perceptions of the extent to which a patient felt safe in their presence and whether he or she felt accepted by them; e.g., ‘My client felt respected by me today’). Higher scores on each subscale (12 items for therapist helpfulness and 10 items for the safe–accepted subscale) represent more desirable outcomes of TR. PEAR‐T has a high reliability score, with Cronbach's alpha ranging from 0.88 to 0.95 (Snyder and Silberschatz 2017). Also, in this study, this scale was characterised by a high reliability score of 0.73 and 0.81 (see Table 2). Beyond internal consistency, the PEAR‐T also provides evidence of construct validity. The scale measures TR directly, rather than inferring it from broader indicators such as overall session satisfaction. It was designed to capture responsiveness from both the therapist's and the patient's perspectives, making it possible to explore this process from either side of the therapeutic relationship. Although therapist and patient ratings of responsiveness are often only modestly related (Snyder and Silberschatz 2017), these viewpoints represent complementary facets of the same process rather than measurement error. This supports the use of the therapist version of the PEAR‐T as a valid tool for examining therapists' self‐perceived responsiveness in psychotherapy research.

**TABLE 2:** Descriptive statistics for analysed variables in the first measurement.

Variable	M	SD	min	max	S	K	alpha
Therapist helpfulness	2.80	0.51	0.92	4.00	−0.39	0.19	0.81
Safe–accepted	2.22	0.25	1.50	3.20	0.15	0.46	0.73
Total responsiveness	2.54	0.33	1.27	3.32	−0.31	0.02	0.84
Depth	4.97	1.00	2.00	7.00	−0.19	−0.34	0.79
Smoothness	4.79	1.02	1.80	7.00	−0.52	−0.20	0.84
Positivity	5.62	0.97	2.40	7.20	−0.59	−0.14	0.84
Arousal	4.13	0.87	1.40	6.60	0.16	0.09	0.65
Satisfaction with life	25.26	4.35	10.00	35.00	−0.58	0.49	0.83
Positive affect	36.03	5.12	18.00	49.00	−0.33	0.32	0.78
Negative affect	18.53	6.25	10.00	42.00	1.01	0.51	0.87

*Note:* min = minimum value; max = maximum value; S = skewness; K = kurtosis; alpha = Cronbach's alpha reliability coefficient.

#### 2.3.2 Session Evaluation Questionnaire

In order to measure how the therapists subjectively evaluated each of the sessions, we utilised a Polish version of the Session Evaluation Questionnaire (SEQ; Stiles et al. 2002). It allows for assessing participants' perceptions of a given session. The scale consists of 21 self‐reported items, which are 7‐point bipolar adjective scales. Opposite adjectives are positioned at the polar ends of the scale. The dimensions measured are *depth* (e.g., shallow–deep, worthless–valuable), *smoothness* (e.g., rough–smooth, tense–relaxed), *positivity* (e.g., sad–happy, afraid–confident) and *arousal* (e.g., still–moving, peaceful–energetic). Scores for each dimension are calculated as the mean of five items, yielding four index scores that range from 1 to 7. Higher scores on each subscale represent more desirable outcomes. There is one additional evaluation item, i.e., bad–good, which is included in the scale for global session evaluation. SEQ has a high reliability score, with Cronbach's alpha ranging from 0.90 to 0.93 (Stiles et al. 2002). In this study, this scale was characterised by a high reliability score ranging from 0.65 to 0.84 for each of the subscales (see Table 2).

#### 2.3.3 SWB

The cognitive evaluation of therapists' SWB was captured through the Polish version of the Satisfaction With Life Scale (SWLS; Diener et al. 1985). It includes five items covering one's declarative life satisfaction. Participants rated statements such as ‘In most ways, my life is close to my ideal or I am satisfied with my life’ on a 7‐point Likert scale ranging from 1 (strongly disagree) to 7 (strongly agree). They were asked to do this task by trying to evaluate how they feel in the particular week. Total scores are calculated by summing the responses to all five items. Higher values reflect greater satisfaction with life.

SWLS has a high reliability score, with Cronbach's alpha estimated at 0.87 (Diener et al. 1985). Also in this study, this tool was characterised by a high reliability score of 0.83 (see Table 2).

The emotional aspect of SWB was assessed via the Polish version of the Positive and Negative Affect Schedule (PANAS; Watson et al. 1988). This 20‐item questionnaire measures both PA and NA. Participants reported how intensely they felt a variety of emotions during the week. Answers are given on a 5‐point Likert scale (1 = very slightly or not at all; 5 = extremely). Separate scores are computed for PA and NA. Higher scores represent stronger experiences of the given affect. PANAS has a high reliability score, with Cronbach's alpha ranging from 0.87 to 0.90 (Watson et al. 1988). Also in this study, PANAS was characterised by a high reliability score of 0.78 for PA and 0.87 for NA (see Table 2).

### 2.4 Data Analysis

First, we examined the sample in terms of sociodemographic and work‐related variables. Next, we analysed the dropout rate and looked into the variables possibly related to dropout with the use of the chi‐square test of independence and Student's *t*‐test for independent samples. The main analysis was performed with generalised estimating equations (GEE). This method enables analysing repeated measurements with interaction effects (Garson 2013). Also, it yields consistent estimates of the regression parameters in case of missing data. It provides tests for statistical significance of changes in time and tests for the statistical significance of interaction effects regarding factors and continuous variables that can affect this change. Statistically significant main effects and interactions are interpreted with the use of parameter estimates. Statistically significant and positive parameter estimates indicate increase and statistically significant and negative parameter estimates indicate decrease. We examined seven dependent variables. Each dependent variable and each factor or continuous variable that potentially could affect the dynamics of change were verified in a separate GEE model. We examined two types of covariates, i.e., the variables that can affect the dynamics of the change in dependent variables. The first type was the static characteristics of therapists, i.e., age, work experience, having completed one's own therapy and therapeutic orientation. We verified between‐subject effects regarding these covariates. We also examined time‐varying covariates, such as current life satisfaction, positive and negative affect. Regarding these covariates, we examined within‐subject effects and whether they covary with how sessions were assessed. The analysis was performed with the use of IBM SPSS Statistics 30.0 software.

## 3 Results

### 3.1 Descriptive Statistics

Table 2 depicts descriptive statistics for the analysed interval variables, i.e., the indicators of therapists' SWB, TR and SET acquired in the first measurement.

The distributions of analysed variables did not deviate from the normal distribution substantially in terms of skewness or kurtosis. They fell within the range of values from −1 to 1. The distribution of NA was positively skewed, meaning that the majority of participants scored low on this scale.

### 3.2 Dropouts

Overall, 232 (out of 296) therapists participated in all 10 measurement waves, which gave us a dropout rate equal to 21.6%. Table 3 presents comparisons of the dropout group and the group of participants who participated in all measurements in terms of sociodemographic and work‐related variables, with statistical significance acquired from Pearson's chi‐square test of independence.

**TABLE 3:** Sociodemographic and work‐related variables of the dropout group and the therapists participating in all measurements.

	Dropout						Dropout				
	No		Yes				No		Yes		
Variable	*n*	%	*n*	%	*p*	Variable	*n*	%	*n*	%	*p*
Gender					0.448	Therapeutic approach					0.169
Female	194	83.6	56	87.5		Humanist‐existential	152	65.5	31	48.4	
Male	38	16.4	8	12.5		Integrative	26	11.2	13	20.3	
Marital status					0.122	Psychodynamic	26	11.2	8	12.5	
Single	25	10.8	13	20.3		Cognitive‐behavioural	10	4.3	5	7.8	
Stable relationship	175	75.4	42	65.6		Psychotraumatology	6	2.6	4	6.3	
Divorced	32	13.8	9	14.1		Systemic	6	2.6	2	3.1	
Education						Other	6	2.6	1	1.6	
Psychology	141	60.8	49	76.6	0.020*	Therapeutic certificate					0.236
Social sciences	45	19.4	9	14.1	0.328	Yes	102	44.0	22	34.4	
Medical or STEM	9	3.9	3	4.7	0.772	In training	123	53.0	38	59.4	
Humanities	30	12.9	1	1.6	0.009*	Not applicable	7	3.0	4	6.3	
Patients						Supervision					0.434
Adults	230	99.1	61	95.3	0.036*	Once a week	78	33.6	17	26.6	
Children and teens	70	30.2	16	25.0	0.420	Once a month	143	61.6	45	70.3	
Couples	54	23.3	15	23.4	0.978	Once a quarter or less	11	4.7	2	3.1	
Families	17	7.3	8	12.5	0.188	Own therapy					0.279
Work status						Yes	166	71.6	51	79.7	
Private practice	224	96.6	60	93.8	0.314	No	5	2.2	0	0	
Public sector	46	19.8	16	25.0	0.368	In therapy	61	26.3	13	20.3	
Volunteering	5	2.2	3	4.7	0.269						
Other	14	6.0	4	6.3	0.949						

*Note: n* = number of participants; % = percentage of the group; *p* = statistical significance in chi‐squared test.

In the dropout group, there were significantly more therapists with a psychological background and significantly fewer therapists with a humanistic educational background. No other associations between dropout and sociodemographic or work‐related variables were statistically significant.

Table 4 presents comparisons of the dropout group and the group of participants who participated in all measurements in terms of the values interval variables acquired in the first measurement, with Student's *t*‐tests for independent samples.

**TABLE 4:** Interval variables of the dropout group and the group participating in all measurements.

	Dropout						
	No		Yes				
Variable	M	SD	M	SD	*t*	*df*	*p*
Therapist helpfulness	2.81	0.52	2.77	0.47	0.48	294	0.315
Safe–accepted	2.22	0.25	2.21	0.26	0.47	294	0.318
Total responsiveness	2.54	0.34	2.52	0.32	0.57	294	0.286
Depth	4.94	1.00	5.09	1.01	−1.05	294	0.148
Smoothness	4.84	1.03	4.63	0.98	1.46	294	0.073
Positivity	5.64	0.99	5.58	0.91	0.46	294	0.324
Arousal	4.11	0.90	4.22	0.74	−0.97	294	0.166
Satisfaction with life	25.67	4.12	23.78	4.83	3.13	294	0.001*
Positive affect	36.23	5.28	35.31	4.42	1.28	294	0.102
Negative affect	18.49	6.39	18.66	5.77	−0.19	294	0.426

*Note: df* = degrees of freedom.

In the dropout group, satisfaction with life was significantly lower than in the group participating in all measurements.

### 3.3 Trajectories of TR

#### 3.3.1 Therapist Helpfulness

**FIGURE 1:**
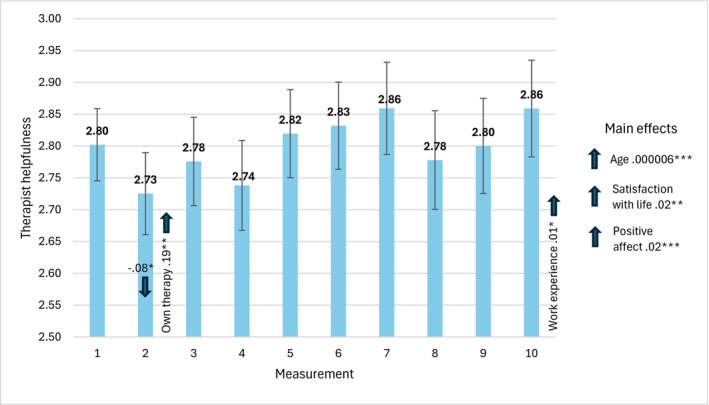
Trajectory of changes in the assessment of perceived therapist helpfulness over 10 weeks. *Note:* The estimates used to interpret statistically significant effects for individual bars are time‐specific. The estimates for main effects, labelled ‘main effects’ are general and not time‐specific.

As mentioned in the Data Analysis section, GEEs yield consistent estimates of the regression parameters in case of missing data. Therefore, the longitudinal analysis below was conducted on the whole sample (*N* = 296). In addition to the main effects, interactions with sociodemographic and work‐related variables were tested in an exploratory manner to examine whether individual and professional characteristics were associated with longitudinal changes in therapists' responsiveness. Figure 1 presents the mean values of therapist helpfulness acquired in 10 consecutive measurements with the values of parameter estimates and 95% CI Wald intervals.

We found a statistically significant effect of change in time, *χ* ^2^(9) = 23.78, *p* = 0.005, a statistically significant interaction effect between completing one's own therapy and change in time, *χ* ^2^(18) = 171.30, *p* = 0.001, a statistically significant interaction effect between work experience and change in time, *χ* ^2^(9) = 31.83, *p* = 0.001, and the main effects of age, *χ* ^2^(1) = 21.65, *p* = 0.001, satisfaction with life, *χ* ^2^(1) = 63.77, *p* = 0.001 and PA, *χ* ^2^(1) = 114.76, *p* = 0.001.

The mean value of therapist helpfulness acquired in the second measurement was significantly lower than the baseline value in the first measurement. In the group of therapists who finished their own therapy, this reduction was not present. Therapists with longer work experience had higher values of therapist helpfulness in the 10th measurement. Also, in general, the older the therapists, the higher the satisfaction with life and PA, and the higher the level of therapist helpfulness.

#### 3.3.2 Safe–Accepted

**FIGURE 2:**
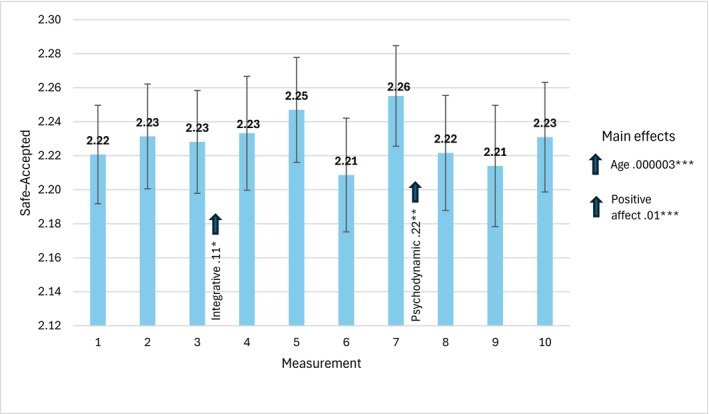
Trajectory of changes in the assessment of perceived safe–accepted over 10 weeks. *Note:* The estimates used to interpret statistically significant effects for individual bars are time‐specific. The estimates for main effects, labelled ‘main effects’ are general and not time‐specific.

Figure 2 presents the mean values of safe–accepted assessment acquired in 10 consecutive measurements, with the values of parameter estimates and 95% CI Wald intervals.

We did not observe a statistically significant effect of change in time, *χ* ^2^(9) = 9.01, *p* = 0.436. However, we found an interaction effect between therapist orientation and change in time, *χ* ^2^(54) = 921.55, *p* = 0.001, and main effects of age, *χ* ^2^(1) = 270.33, *p* = 0.001 and PA, *χ* ^2^(1) = 60.08, *p* = 0.001.

In the group of integrative therapists, an increase in safe–accepted assessment was found in the third measurement, and in the group of psychodynamic therapists in the seventh measurement. In general, the older the therapists and the higher the level of PA, the higher the level of safe–accepted assessment.

#### 3.3.3 Total Responsiveness

**FIGURE 3:**
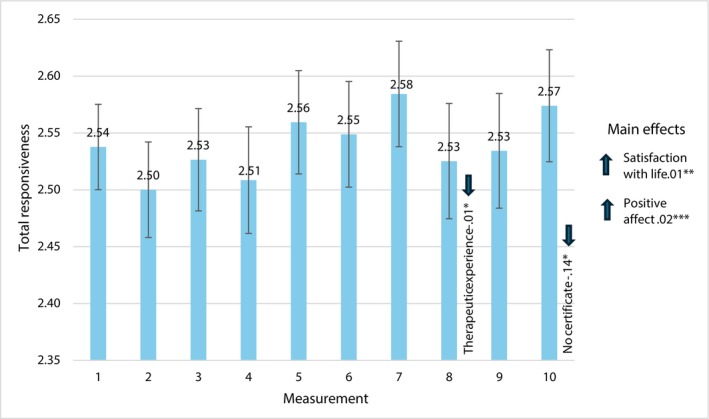
Trajectory of changes in the assessment of perceived total responsiveness over 10 weeks. *Note:* The estimates used to interpret statistically significant effects for individual bars are time‐specific. The estimates for main effects, labelled ‘main effects’ are general and not time‐specific.

Figure 3 presents the mean values of total responsiveness acquired in 10 consecutive measurements with the values of parameter estimates and 95% CI Wald intervals.

We did not find a statistically significant effect of change in time, *χ* ^2^(9) = 17.04, *p* = 0.063. However, we noted interaction effects between having therapeutic certificate and change in time, *χ* ^2^(18) = 51.69, *p* = 0.001 and between work experience and change in time, *χ* ^2^(9) = 26.47, *p* = 0.002, and main effects of satisfaction with life, *χ* ^2^(1) = 61.02, *p* = 0.001 and PA, *χ* ^2^(1) = 124.11, *p* = 0.001.

In the group of therapists without a therapeutic certificate, there was a significant decrease in terms of total responsiveness in the 10th measurement. The therapists with longer work experience had a lower level of total responsiveness in the eighth measurement. In general, the higher the level of satisfaction with life and PA, the higher the level of responsiveness.

### 3.4 Trajectories of Session Evaluation

#### 3.4.1 Depth

**FIGURE 4:**
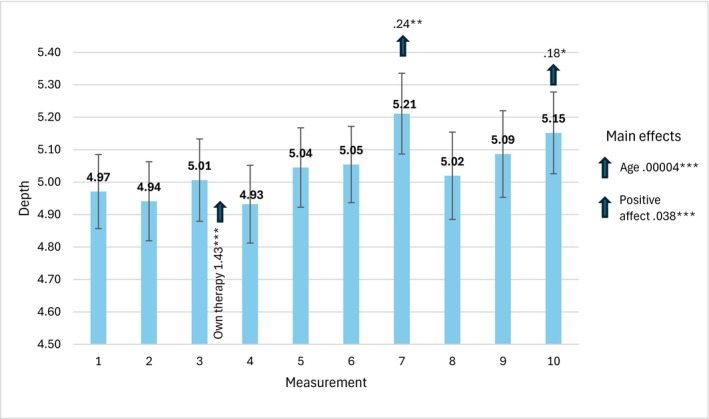
Trajectory of changes in the assessment of perceived depth over 10 weeks. *Note:* The estimates used to interpret statistically significant effects for individual bars are time‐specific. The estimates for main effects, labelled ‘main effects’ are general and not time‐specific.

Figure 4 presents the mean values of depth acquired in 10 consecutive measurements with the values of parameter estimates and 95% CI Wald intervals. Each arrow shows the direction of an effect.

We found a statistically significant effect of change in time, *χ* ^2^(9) = 21.28, *p* = 0.011, a statistically significant interaction effect between completing one's own therapy and change in time, *χ* ^2^(18) = 234.27, *p* = 0.001, and main effects of age, *χ* ^2^(1) = 95.74, *p* = 0.001, and PA, *χ* ^2^(1) = 128.24, *p* = 0.001. The mean values of depth acquired in the 7th and 10th measurements were significantly higher than the baseline value in the first measurement. In the group of therapists who finished their own therapy, the level of depth was significantly higher in the third measurement, so that they achieved a higher level of depth sooner. Also, in general, the older the therapist and the higher the PA level, the higher the level of depth.

#### 3.4.2 Smoothness

**FIGURE 5:**
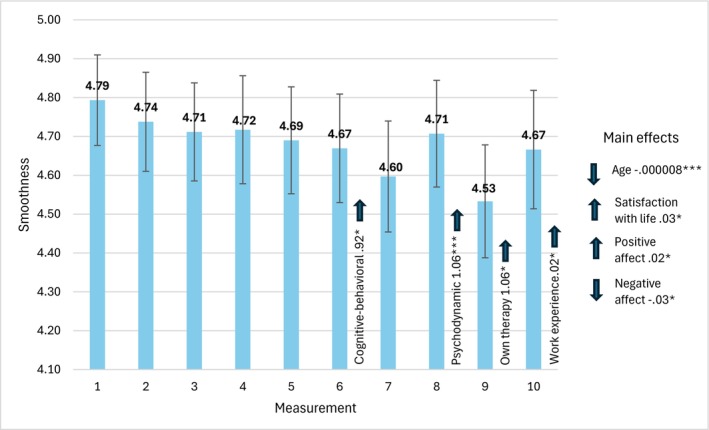
Trajectory of changes in the assessment of perceived smoothness over 10 weeks. *Note:* The estimates used to interpret statistically significant effects for individual bars are time‐specific. The estimates for main effects, labelled ‘main effects’ are general and not time‐specific.

Figure 5 presents the mean values of smoothness acquired in 10 consecutive measurements with the values of parameter estimates and 95% CI Wald intervals.

We did not find a statistically significant effect of change in time, *χ* ^2^(9) = 14.24, *p* = 0.114. However, we found statistically significant interaction effects between one's therapeutic orientation and change in time, *χ* ^2^(54) = 350.57, *p* = 0.001, between completing one's own therapy and change in time, *χ* ^2^(18) = 305.55, *p* = 0.001 and between work experience and change in time, *χ* ^2^(9) = 17.67, *p* = 0.039. Also, we found statistically significant main effects of age, *χ* ^2^(9) = 2737.99, *p* = 0.001, satisfaction with life, *χ* ^2^(1) = 17.25, *p* = 0.001, PA, *χ* ^2^(1) = 34.00, *p* = 0.001 and NA, *χ* ^2^(1) = 36.52, *p* = 0.001.

In the group of cognitive‐behavioural therapists, a significant increase in smoothness was exhibited in measurement no. 6, and in the group of psychodynamic therapists in measurement no. 8. In the group of therapists who finished their own therapy, the level of smoothness was significantly higher in the ninth measurement. The longer the work experience, the higher the probability of increasing smoothness in measurement no. 10. Also, in general, the higher the levels of satisfaction with life and PA, and the lower the level of NA, the higher the level of smoothness.

#### 3.4.3 Positivity

**FIGURE 6:**
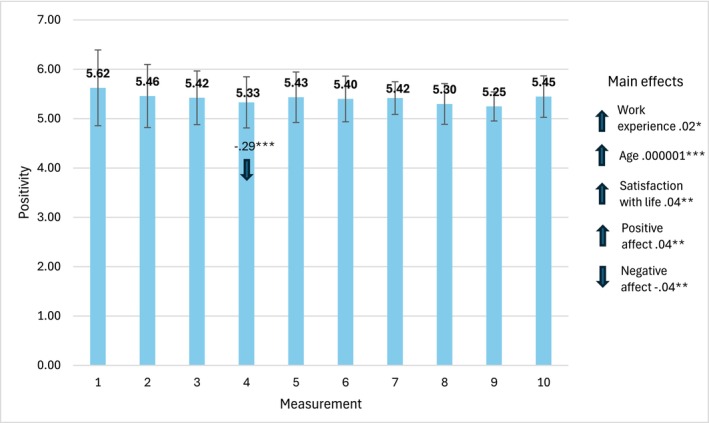
Trajectory of changes in the assessment of perceived positivity over 10 weeks. *Note:* The estimates used to interpret statistically significant effects for individual bars are time‐specific. The estimates for main effects, labelled ‘main effects’ are general and not time‐specific.

Figure 6 presents the mean values of positivity acquired in 10 consecutive measurements with the values of parameter estimates and 95% CI Wald intervals.

We found a statistically significant effect of change in time, *χ* ^2^(9) = 38.84, *p* = 0.001, and main effects of age, *χ* ^2^(1) = 21.07, *p* = 0.001, work experience, *χ* ^2^(1) = 3.81, *p* = 0.050, satisfaction with life, *χ* ^2^(1) = 63.57, *p* = 0.001, PA, *χ* ^2^(9) = 182.80, *p* = 0.001 and NA, *χ* ^2^(1) = 116.50, *p* = 0.001.

The mean value of positivity acquired in the fourth measurement was significantly lower than the baseline value in the first. In general, the longer the work experience and older age, the higher the levels of satisfaction with life and PA, and the lower the level of NA, the higher the level of positivity.

##### 3.4.3.1 Arousal

**FIGURE 7:**
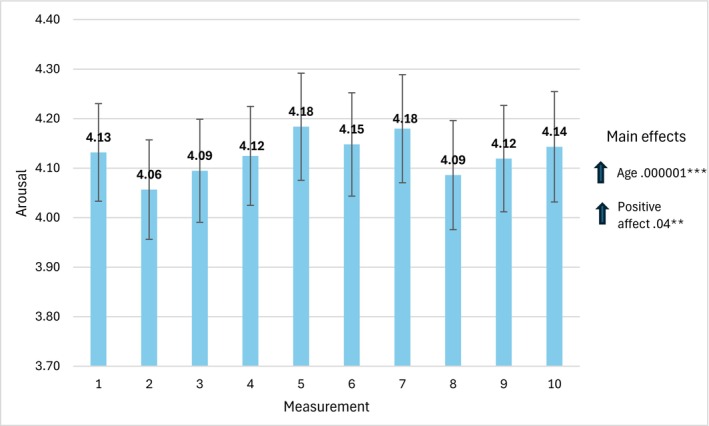
Trajectory of changes in the assessment of perceived arousal over 10 weeks. *Note:* The estimates used to interpret statistically significant effects for individual bars are time‐specific. The estimates for main effects, labelled ‘main effects’ are general and not time‐specific.

Figure 7 presents the mean values of arousal acquired in 10 consecutive measurements with the values of parameter estimates and 95% CI Wald intervals.

We did not observe a statistically significant effect of change in time, *χ* ^2^(9) = 7.22, *p* = 0.614. However, we found main effects of age, *χ* ^2^(1) = 35.19, *p* = 0.001 and PA, *χ* ^2^(9) = 95.63, *p* = 0.001.

In general, the older the therapist and the higher the level of PA, the higher the level of arousal.

### 3.5 Summary

Table 5 summarises the effects detected in the study.

**TABLE 5:** Summary of effects regarding factors impacting TR and its changes.

	SWL	PA	NA	Age	Work exp.	Own therapy	CBT	Psycho‐ dynamic	Integrative	No certificate
Therapist helpfulness	↑	↑		↑	↑(10)	↑(2)				
Safe–accepted		↑		↑				↑(7)	↑(3)	
Total responsiveness	↑	↑			↓(8)					↓(10)
Depth		↑		↑		↑(3)				
Smoothness	↑	↑	↓	↓			↑(6)	↑(8)		
Positivity	↑	↑	↓	↑	↑					
Arousal		↑		↑						

*Note:* SWL, satisfaction with life; PA, positive affect; NA, negative affect; CBT, cognitive‐behavioural therapy; the numbers in brackets relate to a specific number of measurements. Arrows without numbers refer to main effects.

Satisfaction with life was related positively to therapist helpfulness and total responsiveness, as well as to smoothness and positivity levels among participants. PA enhanced all the TR and SET dimensions, while NA hindered smoothness and positivity during the session. Regarding sociodemographic variables, older age was associated with higher therapist helpfulness and safe–accepted and more intense depth, positivity and arousal. At the same time, older therapists were characterised by lower smoothness. Work experience was linked with better therapist helpfulness dynamics and a higher level of positivity. Completing one's own therapy was related to better dynamics regarding therapist helpfulness and depth. Better dynamics of smoothness were detected in the group of cognitive‐behavioural therapists. Better dynamics of smoothness were detected in the group of psychodynamic therapists, and better dynamics of safe–accepted assessment was detected in both the psychodynamic therapists and the integrative therapists. Therapists with no certificate assessed total responsiveness toward the end as being lower.

## 4 Discussion

The results of our study were mostly in line with our hypotheses, as we observed that better SWB of therapists was associated with almost all studied dimensions of their perception of TR and SET during the 10 weeks of observation. More specifically, satisfaction with life was positively related to therapist helpfulness and total responsiveness, as well as to smoothness and positivity levels. In comparison, PA was positively linked to all the TR and SET dimensions, while NA was negatively related to smoothness and positivity in our sample. These findings call for more attention to the therapist's psychological well‐being as significant factor responsible for the therapy process and its outcomes (Laverdière et al. 2018, 2019). They are especially important in light of previous studies focusing mostly on therapists' negative emotional reactions, distress or burnout (e.g., Ackerley et al. 1988; Rosenberg and Pace 2006; Rupert and Morgan 2005; Rzeszutek and Schier 2014). For example, the classic study by Gurman (1972) proved that there is a significant relationship between the therapist's mood and their ability to create a positive therapeutic relationship. Furthermore, Geller et al. (2010) found that daily meditation, as a self‐care practice, positively stimulated the therapeutic relationship quality. In addition, Hayes et al. (2011) reported that therapists' self‐care practices enhancing general life satisfaction and quality of life, such as resting and exercising, reduced the prevalence and intensity of countertransference behaviours. More recently, Chui et al. (2016) concluded that the therapist's affective well‐being may independently contribute to the process, as well as the outcomes, of therapy sessions. The potential mechanism linking therapist well‐being and psychotherapy outcomes is probably related to clients' perceptions of therapists' behaviours and the overall therapeutic relationship. Therapists who appear distressed are viewed as being more disrespectful, judgmental and uninterested in forming a genuine connection. This can hinder the therapeutic alliance over time (Vybíral et al. 2024). More broadly, therapist life satisfaction may reflect underlying psychological resources such as emotional stability, resilience and lower emotional exhaustion, which can enhance therapists' presence, empathic attunement and consistency within sessions. Therapists with higher life satisfaction may be better able to regulate countertransference reactions, remain emotionally available and convey hope and authenticity—qualities that clients may perceive as safety and engagement, thereby strengthening the therapeutic process. Conversely, lower life satisfaction may subtly affect affective tone, energy levels or interpersonal sensitivity, shaping session dynamics even when technical competence remains unchanged. However, this topic requires further elaboration in future studies. Specifically, therapists' ability to mentalise their own emotions may be crucial for greater depth of in‐session exploration and the capacity for resolving in‐session ruptures, allowing for a better working alliance over time (Reading et al. 2019).

We also observed interesting associations between sociodemographic and work‐related variables in the dynamics of TR and SET among participants. Older age, greater work experience, finishing one's own therapy, as well as having a professional certificate usually predicted greater TR and better SET and were linked with higher satisfaction with life and better affective well‐being. These findings are in line with some reporting that older therapists experience higher levels of psychological well‐being (Brugnera et al. 2020) and suffer less from work‐related stress (e.g., burnout; Van Hoy et al. 2023a) than their younger colleagues. Alternatively, however, the significant effect of age may partly also reflect biased self‐perception among older therapists and/or reduced critical self‐scrutiny. In addition, we found that greater work experience was associated with occasional decreases in responsiveness, as well as increases in perceived therapist helpfulness. These results suggest that work experience plays a nuanced role in shaping session evaluations. On the one hand, experienced therapists may view their work as more positive and satisfying (Stein and Lambert 1995); on the other, they may also become less attuned at times, possibly due to routinisation, a decline in reflective practice or emotional fatigue (Goldberg et al. 2016). Furthermore, our study provided some evidence for the role of therapist personal therapy in enhancing therapists' well‐being (e.g., Orlinsky et al. 2005, 2011). Nevertheless, this finding needs to be treated with caution, as we did not examine the specific effect of personal therapy, so our observations may remain somehow speculative. Finally, in terms of differences between therapeutic modalities, we noticed different patterns of changes between cognitive‐behavioural, psychodynamic and integrative therapists. Psychodynamic therapists demonstrated a more rapid increase in session depth compared to other modalities. Cognitive‐behavioural therapists scored higher on session smoothness; however, unlike other modalities, they did not experience a significant increase in session depth toward the end of the study. Therapists practicing within an integrative framework had higher ratings in the safe–accepted domain, which indicates an increased focus on clients' experiences of comfort and acceptance. These patterns highlight clear differences in psychotherapeutic approaches and echo recent research on therapeutic responsiveness in different models indicating that responsiveness is an extra‐specific factor expressed uniquely within each modality (Davì et al. 2024).

### 4.1 Strengths and Limitations

This study has several strengths, including the theoretically driven and longitudinal methodological design with innovative data analysis with a large sample of therapists. However, several limitations should also be mentioned. First, in our study, we focused only on therapists and client opinions were not taken into account. Including the clients in this demanding project could have introduced significant organisational and ethical constraints and might have affected the answers of the therapists, as they could have felt ‘judged’ by their clients (possibly leading to a social desirability bias). Nevertheless, the absence of client perspectives may substantially limit our study conclusions and should be taken into account in future studies on this topic. Furthermore, as the study sample probably included mostly therapist–client dyads with an established alliance, the results may differ for dyads where such an alliance has not yet been formed. Second, the study was based on self‐reported measures, with possible retrospective bias that may have affected the results obtained in this study. Third, due to organisational constraints, the study participants self‐selected their clients, which might have introduced bias toward including cases that therapists believed were likely to showcase good TR or SET. Moreover, due to organisational reasons, therapists were instructed to refer to the most recent session with this client even if the session did not occur that week. Fourth, while GEE is not an inherently causal inference method, it can be adapted for this purpose if it relies on specific assumptions about the data. In our study, we assumed that the therapist's well‐being, including their general perspective on life satisfaction and mood, affects individual therapeutic sessions, rather than the other way around. We acknowledge that many challenging sessions can impact the therapists' well‐being as well, which may be an interesting topic to explore in future studies. Finally, our sample was a convenience sample of Polish therapists (indicating possible selection bias) with limited control over confounding variables, and thus cannot be considered representative of Polish therapists in general.

### 4.2 Clinical Implications

Despite these limitations, our study calls for a greater focus on the psychological well‐being of therapists, which is crucial for their mental health per se and may also be associated with the quality of their work with clients (Laverdière et al. 2018, 2019). It is particularly related to therapists' emotional functioning, which may be related to the therapy process over time (Abargil et al. 2025). From a practical point of view, therapists should be more cautious of their emotional well‐being. Regular supervision can help therapists concentrate on different ways in which their emotions impact their selection of specific interventions in their clinical work. Overall, there is a need for more programs for therapists on how they can cope with work stress by focusing on their self‐care behaviours, which may help them balance their professional and private lives and, as a consequence, boost satisfaction from this occupation and avoid potential burnout (Rupert and Dorociak 2019; Van Hoy and Rzeszutek 2022).

## Funding

The authors have nothing to report.

## Ethics Statement

All the participants provided informed consent. The project was approved by the ethics committee of the Faculty of Psychology at the University of Warsaw.

## Conflicts of Interest

The authors declare no conflicts of interest.

## Data Availability

All the data are available upon request from the corresponding author.
